# The Effects of Polyhexamethylene Biguanide (PHMB) and TLR Agonists Alone or as Polyplex Nanoparticles against *Leishmania infantum* Promastigotes and Amastigotes

**DOI:** 10.3390/vetsci7040179

**Published:** 2020-11-13

**Authors:** Pamela Martínez-Orellana, Marta Baxarias, Liam Good, Laia Solano-Gallego

**Affiliations:** 1 Departament de Medicina i Cirurgia Animals, Facultat de Veterinària, Universitat Autònoma de Barcelona, Bellaterra, 08193 Barcelona, Spain; pamela.martinez.phd@gmail.com (P.M.-O.); marta.baxarias@uab.cat (M.B.); 2 Department of Pathobiology and Population Sciences, The Royal Veterinary College, London NW1 0NH, UK; lgood@rvc.ac.uk

**Keywords:** TLR4 agonist, TLR9 agonist, PHMB, *Leishmania infantum*, canine, macrophages

## Abstract

Dogs are the main reservoir for *Leishmania infantum,* manifesting from a subclinical to a fatal disease. Limited treatments are available, although new antiparasitics and immunomodulators are pursued. Polyhexamethylene biguanide (PHMB) has a broad antimicrobial spectrum, including antiparasitic activity. Here, we evaluated the potential for Toll-like receptor agonists (TLRa) and PHMB alone, and as polyplex nanoparticles containing PHMB and TLR4 or TLR9 agonists, to selectively kill *L. infantum*. Susceptibility of *L. infantum* promastigotes to PHMB, miltefosine, and allopurinol was performed, and the half-maximum inhibitory concentrations (IC_50_) were determined. Then, DH-82 cells were infected and treated with PHMB alone or combined with TLR4a (MPLA-SM) or TLR9a (CpG ODNs) and allopurinol alone. The IC_50_ values of *L. infantum* promastigotes were PHMB (1.495 µM), miltefosine (9.455 µM), and allopurinol (0.124 µM). After infection, treated DH-82 cells displayed a lower percentage (*p =* 0.0316), intensity (*p =* 0.0002), and index of infection (*p =* 0.0022) when compared to non-treated cells. PHMB induced lower percentage of infection alone (*p =* 0.043), in combination with TLR9a (*p =* 0.043), and with TLR4a (*p =* 0.0213). Supernatants were collected and used to measure TNF-α and IL-6 levels. Increased TNF-α was observed after PHMB *plus* TLR4a, relative to uninfected and infected untreated macrophages (*p =* 0.043). PHMB combined with TLR4a shows promise as a potential anti-*L. infantum* drug combination, as well as inducer of proinflammatory response, as demonstrated by decreased infection and increased TNF-α production.

## 1. Introduction

Leishmaniases are a group of vector-borne infectious diseases in humans and animals caused by different species of *Leishmania* [1,2]. The genus *Leishmania* has a digenetic life cycle with two distinct stages recognized. An elongated shape and long flagella characterize the promastigote stage. Promastigotes are further subdivided into procyclic promastigotes that multiply in the gut of blood feeding sandfly phlebotomine and infective metacyclic promastigotes, housed in the mouth and anterior gut of the sand fly vector [3]. Later in the lifecycle, the parasite continues to differentiate in the mammalian host as a non-motile amastigote with rounded or oval shaped cell that lacks flagella. The amastigote stage commonly resides and replicates within the phagolysosome of macrophages [4].

Canine leishmaniosis (CanL) is endemic in many countries of the Mediterranean basin [5]. Domestic dogs are the principal reservoir of *Leishmania infantum* with a high prevalence of infection [5]. CanL is a zoonotic infectious disease with a wide range of clinical manifestations [6,7]. The development of subclinical or clinical states depends on the potential of the humoral and cell-mediated immune responses to self-eliminate the parasite [8]. The conventional treatment strategies used to control the infection require a long period of sustained treatment, do not guarantee a complete recovery, and do not contain spread [6,9]. Furthermore, most of the current therapeutic strategies can be associated with adverse side effects [10,11,12] and do not reinforce the immune system. 

It is important to highlight that the failure of conventional treatments for *Leishmania* infection is increasing [10,13]. In many cases, drug resistance is responsible for non-successful treatment [13]. However, it is well known that other aspects such as the immunological characteristics of the host, the quality and pharmacokinetics of the drug used, as well as the parasite features or eventual co-infections are also involved [14]. Therefore, identification of new drug strategies and immunomodulators that can help to reduce the length of *Leishmania* treatments and improve efficacy to combat this zoonotic infection are very important.

Macrophages are targeted host cells for *L. infantum* infection; even so, they are also capable of antigen-presentation and killing intracellular parasites by reactive oxygen and nitrogen intermediates mechanisms which is an important instrument of response against this infection [15,16,17]. However, *Leishmania* parasites evade the innate immune system by several mechanisms, such as the ability to survive and replicate within macrophage phagolysosomes by producing compounds such as lipophosphoglycans (LPG). The LPG inhibits phagosome maturation [18] and the interference of signaling pathways [19]. 

Isolation, culture, and characterization of canine macrophage cell lines derived from malignant histiocytosis have been described [16]. The mechanisms and receptor-ligands implicated in the interaction, attachment, and uptake of *Leishmania* promastigotes by macrophages have been previously investigated [20]. Therefore, the use of canine macrophage cell lines for functional studies is a very attractive model [21], as it requires a less invasive approach for obtaining the cells comparing to peritoneal or bone marrow-derived macrophages. Such welfare issues are becoming better appreciated when designing experiments in which animals are studied [22]. 

The effect of stimulation of macrophages receptors such as Toll-like receptors (TLRs) during *L. infantum* infection has been little studied (see in [18] for a review). TLRs are the crucial regulators of innate immune responses against various pathogens including *Leishmania* parasites. They are located on the plasma membrane or internal membranes of macrophages, dendritic cells (DCs), natural killer (NK) cells and T and B lymphocytes [23]. TLRs are involved in a variety of phenomena including phagocytosis, maturation, microbicidal activity, and production of cytokines [24]. Once the different TLRs are stimulated by specific ligands, a cascade that induces the production of proinflammatory cytokines such as interleukin (IL)-1α, IL-6, IL-12, tumor necrosis factor-alpha (TNF-α), and interferon-gamma (IFN-γ) is activated [25,26]. Several TLRs play important roles in pathogenesis, susceptibility and resistance to control *Leishmania* infections [27,28]. TLR4 contributes to control the growth of *Leishmania* in innate and adaptative immune responses. It is also a strong regulator of inducible nitric oxide synthase (iNOS) leading to death of the parasite [29]. TLR9 is required for neutrophil recruitment and DCs activation during *Leishmania* infection [30]. However, the spectrum and specificity of how *Leishmania* interaction with TLR agonists (TLRa) induces proinflammatory cytokines is complex and not well established [18] and the mechanisms by which macrophages kill *Leishmania* in dogs have not fully been elucidated [31].

Polyhexamethylene biguanide (PHMB) is a polymer composed of repeating basic biguanidine units connected by hexamethylene hydrocarbon chains, which provide a cationic and amphipathic structure [32]. PHMB has broad antimicrobial spectrum. It is used to treat certain parasite infections and it is widely used in wound care applications [33]. PHMB’s leishmanicidal mechanisms of action appear to involve disruption of parasite cell membranes, cell entry, and condensation/disruption of parasite chromosomes [34]. Nevertheless, the leishmanicidal effect and mechanisms of PHMB has been investigated only in *Leishmania major* demonstrating the killing of promastigotes at low concentrations [34]; however, studies related to others *Leishmania* species are lacking.

In order to elucidate the *in vitro* antileishmanial properties of PHMB against *L. infantum*, here we studied the infection profile and proinflammatory cytokines produced by infected DH-82 cells after treatment with PHMB alone or as polyplex nanoparticles containing TLR4 or TLR9 agonists (TLR4a and TLR9a). These studies may underpin the development of new antimicrobial and immunomodulatory strategies for the control of human and animal leishmaniosis.

## 2. Materials and Methods 

### 2.1. Leishmania infantum Parasite Maintenance

*Leishmania infantum* (MCAN/ES/92/BCN-83) was used in this experiment. Parasites were cultured in biphasic Novy-MacNeal-Nicolle (NNN) medium and sub-passaged in Schneider’s *Drosophila* medium (Biowest^®^, Riverside, MO, USA) supplemented with 20% of fetal bovine serum ((FBS) Biowest^®^, Riverside, MO, USA), 100 U/mL of penicillin (Life Technologies^TM^, Carlsbad, CA, USA), and 1% of filtrated human urine from a healthy donor.

### 2.2. Macrophages Maintenance

The canine macrophage-derived cell line DH-82 (ATCC^®^ CRL-10389™, USA), from a dog with malignant histiocytosis, was maintained in Roswell park memorial institute (RPMI) 1640 medium (Biowest^®,^ Riverside, MO, USA) with 10% of inactivated FBS Premium (Biowest^®^**,** Riverside, MO, USA) supplemented with 100 U/mL of penicillin, 100 µg/mL streptomycin (Life Technologies^TM^, California, USA), and 2 mM L-glutamine (Biowest^®^, Riverside, MO, USA) at 37 °C in 5% CO_2_–95% air. 

### 2.3. Drugs and TLR Agonists

Allopurinol (Sigma-Aldrich, St. Louis, MO, USA) and miltefosine (10 mg/mL, Sigma-Aldrich, MO, USA) were used. Stock final solutions were prepared in ultrapure water for allopurinol (5 mg/mL) and miltefosine (100 µg/mL). TLR4a (monophosphoryl lipid A from *Salmonella minnesota*; [MPLA-SM], Invivogen, San Diego, CA, USA) and TLR9a (5′tccatgacgttcctgatgct CpG oligodeoxynucleotides (CpG ODNs) Invivogen, San Diego, CA, USA) were tested alone and as a polyplex nanoparticles with PHMB (stock concentration of 1 mg/mL). All drugs and TLRa solutions were stored at 4 °C until used.

### 2.4. Preparation and Analyses of Polyplex Nanoparticles

The nanoparticles were prepared in aqueous solution by nanoprecipitation within 2 mL polypropylene microfuge tubes [34]. The final ligand and polymer concentrations are listed in Table 1. First, ligand solutions (TLR4 ligand: MPLA-SM in 30% ethanol and TLR9 ligand: CpG ODNs in water) were diluted in water to the 2 x concentrations indicated in Table 1 and to a final volume of 200 μL, followed by addition of 200 μL of PHMB solution; the mixtures were then stirred for 20 min. After preparation, nanoparticle populations were subjected to dynamic light scattering (DLS) measurements to characterize particle populations. Particle sizes and zeta potential of ligand:PHMB polyplexes were measured by using a Zetasizer Nano S-90 (Malvern Panalytical Ltd., Malvern, UK). The formulations were performed in triplicate. 

### 2.5. Half Inhibitory Concentration (IC_50_) Promastigote Susceptibility

Promastigote drug susceptibility was determined by using AlamarBlue^®^ cell viability reagent (10% *v*/*v*, Life Technologies^TM^, Carlsbad, CA, USA) resazurin reaction. *Leishmania infantum* promastigotes in the stationary phase of *in vitro* growth were cultured at a concentration of (1 × 10^5^ parasite/well) in 100 µL each well in flat-bottomed 96-well cell culture microtiter plates (Costar, Corning, New York, NY, USA). Briefly, starting solutions for allopurinol (2.5 µg/mL, Sigma-Aldrich, St. Louis, MO, USA), miltefosine (50 µg/mL, Sigma-Aldrich, St. Louis, MO, USA), and PHMB (10 µg/mL) were subjected to 2-fold serial dilution until a total of 10 dilutions for each drug. Then, 100 µL of each dilution were added to the promastigotes and incubated at 26 °C for 24 h. Finally, 20 μL of AlamarBlue^®^ (10% *v*/*v*) was added and the cultures were kept for 24 h for drug susceptibility determination following the manufacturer’s recommendations by reading the absorbance at 540 and 590 nm in a Fluoroskan fluorimeter (ThermoLabsystems, Helsinki, Finland). Relative viability was calculated from the ratio of the optical density (OD) results in parasites exposed to the drugs *versus* those not exposed. For each drug, three independent and reproducible experiments were performed in triplicate (total 9 wells for each dilution), drug concentrations alone and parasites alone were added as controls to each plate to calculate the inhibitory concentrations that kill 50% of *L. infantum* promastigotes (IC_50_). 

### 2.6. Cytotoxicity for DH-82 Cell Line

DH-82 cells in the logarithmic phase of growth were counted in a Neubauer chamber, seeded in a 96-well culture plate (Costar, Corning, NY, USA) at a concentration 1 × 10^5^ cells in 100 µL each well in complete RPMI and incubated in an atmosphere of 5% CO_2_ at 37 °C. After 24 h, supernatant was gently removed and two washes with 100 µL each of Dulbecco’s phosphate-buffered saline (DPBS) were made, then 100 µL of fresh media was added to the cells. Dilutions of allopurinol, miltefosine, and PHMB were performed as described above and tested on DH-82 cells. After preparation of 2-fold serial dilutions in complete RPMI, 100 µL of each drug was added to the cells and incubated in an atmosphere of 5% CO_2_ at 37 °C for 24 h. Then, 20 μL of AlamarBlue^®^ (10% *v/v*) was added and the cultures were kept for 24 h and absorbance was read as described above for promastigotes. For each drug, three independent and reproducible experiments were performed. For each drug, three independent and reproducible experiments were performed in triplicate (total 9 wells for each dilution), drugs concentrations aloneand parasites alone were added as controls to each plate to calculate the inhibitory concentrations that kill 50% of DH-82 cells (IC_50_). 

### 2.7. Intracellular L. infantum Amastigotes Susceptibility Assay

Macrophages (5 × 10^4^ cells/well) were incubated in 8 wells (Lab-Tek^®^ Chamber Slide™ (Thermo Scientific Nunc^®^, Waltham, MA, USA)) in 200 µL of complete RPMI medium for 24 h. Then, the medium was removed and DH-82 cells were resuspended with 200 µL of complete RPMI containing promastigotes (stationary phase) at a 10:1 parasite:host cell ratio. The cultures were incubated at 37 °C in 5% CO_2_–95% ambient air for 3 h and then infected DH-82 cells were washed two times with DPBS (Biowest^®^, Riverside, MO, USA) prior to addition of the different conditions. Conditions were prepared as follows. (1) PHMB alone (stock concentration of 1 mg/mL), at a maximum dose tolerated by DH-82 cells (6.133 µM). (2) TLR4a alone ([MPLA-SM]: 0.092 mg/mL stock solution, Invivogen^®^, San Diego, CA, USA) at a final concentration of 1 µg/mL. (3) TLR9a alone ((CpG ODNs) 0.092 mg/mL stock solution, Invivogen^®^, San Diego, CA, USA) at a final concentration of 1 µg/mL. (4) PHMB associated with TLR4a (0.092 mg/mL stock solution) at a final concentration of 1 µg/mL. (5) PHMB associated with TLR9a (0.092 mg/mL stock solution) at a use concentration of 1 µg/mL. (6) Allopurinol alone (stock concentrations of 50 mg/mL), maximum tolerated dose 0.124 µM for allopurinol. Cytotoxicity of each studied drug for DH-82 cells was previously determined. Also, DH-82 cytotoxicity was determined for TLR agonists alone and for combination with PHMB + TLR agonist compounds (data not shown). All control uninfected conditions were also included. Five independent assays with four replicates per each condition were performed. At 24 h, supernatants were collected and stored at −80 °C and Lab-Tek^®^ Chamber Slide™ were fixed with methanol and Diff-quick stained (QCA, Amposta, Tarragona, Spain).

### 2.8. Percentage of L. infantum-Infected Macrophages, Intensity of Infection and Infection Index

The percentage of infected cells (i.e., number of infected cells per 100 macrophages) and the intensity of infection (i.e., number of intracellular amastigotes per number of infected macrophages) were determined by light microscopy under oil (×1000) by counting at least 300 cells per well. Additionally, the infection index (II) was obtained as previously reported multiplying the percentage of infected macrophages and the number of internalized amastigotes per infected macrophage [35]. 

### 2.9. Sandwich ELISA for the Determination of TNF-α and IL-6

Cytokine analysis of TNF-α and IL-6 were performed according to manufacturer’s instructions, (DuoSet^®^ ELISA by Development System R&D TM, Abingdon, UK) using a 96-well cell plate flat bottom (Costar ^®^ Corning, USA). Standard curve for TNF-α started with 1000 pg/mL and twofold dilutions were made until 15.63 pg/mL concentration. Standard curve for IL-6 started with 4000 pg/mL and twofold dilutions were made until 62.5 pg/mL concentration. Optical density was measured with an ELISA reader (Anthos 2020, UK) at wavelength of 450 nm. The standard curve for each cytokine was calculated using a computer generated four parameter logistic curve-fit with program myassays (http://www.myassays.com/). Plate was repeated when R^2^-value of standard curve was below 0.98.

### 2.10. Statistical Analysis

Data were analyzed with one-way ANOVA and post hoc Dunnett’s multiple comparisons test to compare every treatment mean with the control uninfected macrophages mean. Then, a nonparametric Wilcoxon signed rank test was used to compare among related several treatments. 

The statistical analysis in the case of Wilcoxon signed rank test was performed using the SPSS 17.0 for Windows software (SPSS Inc., Chicago, IL, USA), ANOVA and post hoc Dunnett’s test were performed using GraphPad Prism 6 (GraphPad Software, La Jolla, CA, USA). Differences were considered significant with a 5% significance level (*p* < 0.05). All graphs were performed using excel GraphPad Prism 6 (GraphPad Software, La Jolla, CA, USA).

## 3. Results

### 3.1. Polyplex Nanoparticles 

To complex TLR ligand into polyplex nanoparticles, we used nanoprecipitation with the cationic polymer PHMB. Aqueous solutions of two TLR ligands were separately prepared and complex with an aqueous solution of polymer and polyplex nanoparticle populations were profiled by DLS. Values for size and polydispersity are indicated in Table 1. Based on the particle population results, low dispersity formulations for TLR4 and TLR9 ligands were selected for cell studies. 

### 3.2. Promastigotes and Macrophage Drugs Susceptibility

Drug susceptibility of *L. infantum* promastigotes and DH-82 canine macrophages were performed in separate experiments. The IC_50_ values for allopurinol, miltefosine, and PHMB were calculated and then summarized in Table 2. 

### 3.3. Percentages of Infected Macrophages 

**Figure 1:**
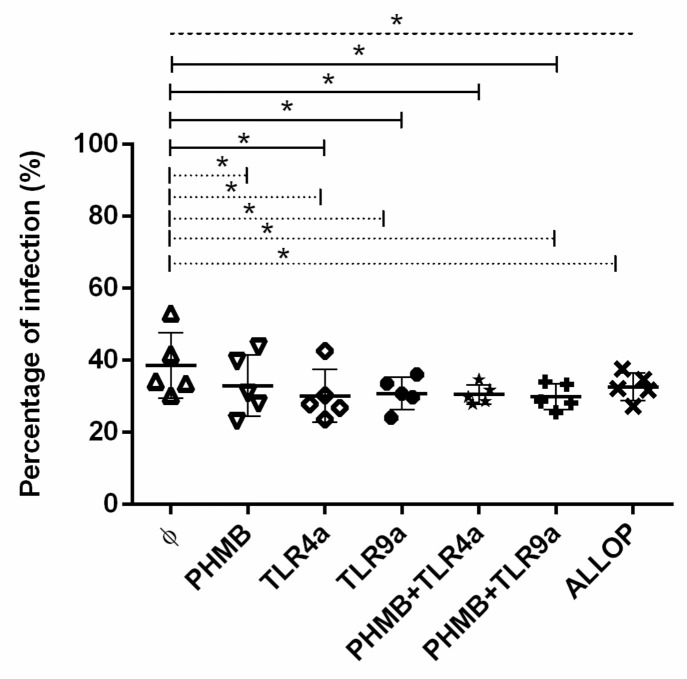
Percentage of *in vitro L. infantum* infected macrophages after 24 h treatment with the different conditions: Medium alone (Ø), Polyhexamethylene biguanide alone (PHMB), MPLA Toll-like receptor agonist 4 (TLR4a), PHMB *plus* TLR4a (PHMB + TLR4a), CpG ODN Toll-like receptor agonist 9 (TLR9a), PHMB *plus* TLR9a (PHMB + TLR9a), and allopurinol (ALLOP). Significant results are described as * 0.05 > *p* > 0.01. Lines in graphic represent ANOVA (dashed lines), Dunnett’s test (solid lines), and Wilcoxon signed-rank test (dotted lines).

To determine the infectivity degree, the mean ± standard deviation (mean ± sd) of the percentage of infected macrophages was calculated and plotted in Figure 1. Infected macrophages without any treatment displayed a range of infection within 30% to 53% with a mean ± sd of 38.5 ± 8.0%. As expected, infected macrophages alone showed the higher percentage of infection when compared to all treatment conditions studied (ANOVA: *p =* 0.0316). Infected macrophages that were un-treated showed a higher level of infection when compared with infected cells treated with TLR4a alone (Dunnett’s test: *p =* 0.0154), TLR9a alone (Dunnett’s test: *p =* 0.0269), PHMB *plus* TLR4a (Dunnett’s test: *p =* 0.0213), and PHMB *plus* TLR9a (Dunnett’s test: *p =* 0.0121).

Significantly higher percentage of infection was detected when untreated infected macrophages were compared to infected cells treated with PHMB alone (% mean ± sd of 34.7 ± 6), TLR4a alone (% mean ± sd of 30.4 ± 6.4), TLR9a alone (% mean ± sd of 32.7 ± 2.1), PHMB *plus* TLR9a (% mean ± sd of 30.8 ± 3.2), and allopurinol (% mean ± sd of 30.9 ± 5) alone (Wilcoxon signed-rank test: *p =* 0.043). A tendency towards higher percentage of infection was noticed when infected macrophages without treatment were compared with macrophages treated with PHMB *plus* TLR4a (Wilcoxon signed-rank test: *p =* 0.08). Additionally, no differences were detected when PHMB alone was compared with the rest of the conditions studied. Moreover, no differences were found when comparing TLR4a (% mean ± sd of 30.4 ± 6.4) or TLR9a (% mean ± sd of 32.7 ± 2.5) alone with PHMB *plus* TLR4a (% mean ± sd of 28.4 ± 2.5) or PHMB *plus* TLR9a (% mean ± sd of 30.8 ± 3.2) (Figure 1). 

### 3.4. Intensity of Infection

**Figure 2:**
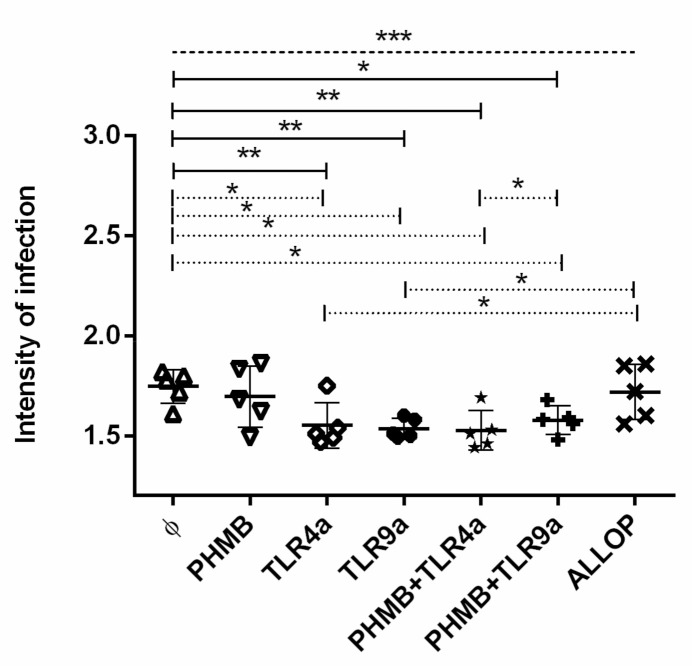
Intensity of *in vitro L. infantum* macrophage infection after 24 h treatment with the different conditions: Medium alone (Ø), Polyhexamethylene biguanide alone (PHMB), MPLA Toll-like receptor agonist 4 (TLR4a), PHMB *plus* TLR4a (PHMB + TLR4a), CpG ODN Toll-like receptor agonist 9 (TLR9a), PHMB *plus* TLR9a (PHMB + TLR9a), and allopurinol (ALLOP). Significant results are described as * 0.05 > *p* > 0.01, ** 0.01 > *p* > 0.001, *** 0.001 > *p* > 0.0001. Lines in graphic represent ANOVA (dashed lines), Dunnett´s test (solid lines), and Wilcoxon signed-rank test (dotted lines).

The intensities of infection were analyzed, and the result of all experiments is displayed in Figure 2. Statistically significant differences were observed when infected macrophages alone were compared to the rest of the treatment conditions (ANOVA: *p =* 0.0002). Particularly, infected macrophages treated with TLR4a alone (Dunnett’s test: *p =* 0.0040), TLR9a alone (Dunnett’s test: *p =* 0.0018), PHMB *plus* TLR4a (Dunnett’s test: *p =* 0.0011) and PHMB *plus* TLR9a (Dunnett’s test: *p =* 0.0138) showed lower intensity of infection than infected non-treated cells. A lower number of amastigotes were observed in macrophages treated with TLR4a (mean ± sd of 1.56 ± 0.0) and TLR9a (mean ± sd of 1.53 ± 0.0), PHMB *plus* TLR4a (mean ± sd of 1.50 ± 0.0), and PHMB *plus* TLR9a (mean ± sd of 1.57 ± 0.0) when compared to infected macrophages without treatment (Wilcoxon signed-rank test: *p =* 0.043). Additionally, macrophages treated with TLR4a or TLR9a alone showed lower intensity of infection than macrophages treated with allopurinol (Wilcoxon signed-rank test: *p =* 0.043) (Figure 2). In addition, a lower significantly intensity of infection was observed on cells treated with PHMB *plus* TLR4a when compared to PHMB *plus* TLR9a treatment (Wilcoxon signed-rank test: *p =* 0.043) (Figure 2).

Finally, a tendency towards higher intensity of infection was noticed when infected macrophages without treatment were compared with macrophages treated with PHMB alone (Wilcoxon signed-rank test: *p =* 0.08) as well as when infected macrophages treated with PHMB *plus* TLR4a were compared to infected macrophages only treated with TLR4a alone (Wilcoxon signed-rank test: *p =* 0.08).

### 3.5. Infectivity Index 

**Figure 3:**
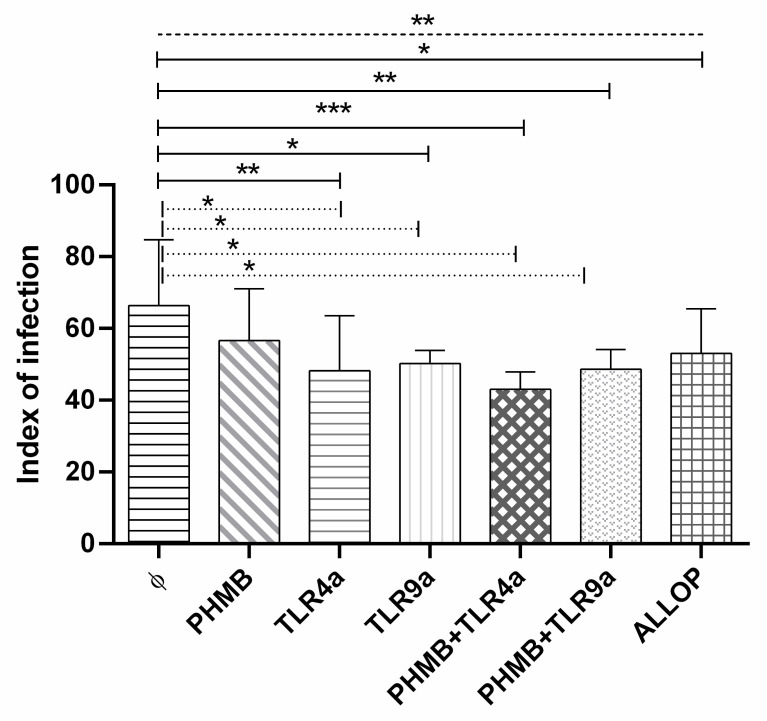
Infection index of *in vitro L. infantum* macrophage infection after 24 h treatment with the different conditions: Medium alone (Ø), Polyhexamethylene biguanide alone (PHMB), MPLA Toll-like receptor agonist 4 (TLR4a), PHMB *plus* TLR4a (PHMB + TLR4a), CpG ODN Toll-like receptor agonist 9 (TLR9a), PHMB *plus* TLR9a (PHMB + TLR9a), and allopurinol (ALLOP). Significant results are described as * 0.05 > *p* > 0.01, ** 0.01 > *p* > 0.001, *** 0.001 > *p* > 0.0001. Lines in graphic represent ANOVA (dashed lines), Dunnett´s test (solid lines), and Wilcoxon signed-rank test (dotted lines).

At 24 h post-infection, we compared the infectivity index of the macrophages under different conditions (Figure 3). As previously observed, a higher infection index was achieved in non-treated macrophages (mean ± sd of 66.4 ± 16.1) when compared to the rest of the treatments (ANOVA: *p =* 0.0022). Differences were found with TLR4a alone (Dunnett’s test: *p =* 0.0048), TLR9a alone (Dunnett’s test: *p =* 0.0129), PHMB *plus* TLR4a (Dunnett’s test: *p =* 0.0004) and PHMB *plus* TLR9a (Dunnett’s test: *p =* 0.0058), and allopurinol (Dunnett’s test: *p =* 0.0479) when compared with un-treated macrophages. Furthermore, lower but not significantly differences (Dunnett’s test: *p =* 0.2103) were found when PHMB alone (mean ± sd of 56.3 ± 13.0) was compared with macrophages without treatment. 

A lower infection index was found in the infected macrophages treated with PHMB *plus* TLR4a (mean ± sd of 42.9 ± 4.2, Wilcoxon signed-rank test: *p =* 0.043), and PHMB *plus* TLR9a (mean ± sd of 48.5 ± 4.9, Wilcoxon signed-rank test: *p =* 0.042) and also in TLRa treatments alone, TLR4a (mean ± sd of 48.2 ± 13.4, Wilcoxon signed-rank test: *p =* 0.041) and TLR9a (mean ± sd of 50.1 ± 3.3, Wilcoxon signed-rank test: *p =* 0.042) when compared with non-treated infected macrophages (Figure 3).

### 3.6. TNF-α and IL-6 Concentrations on Supernatant of DH-82 Treated Cells

**Figure 4:**
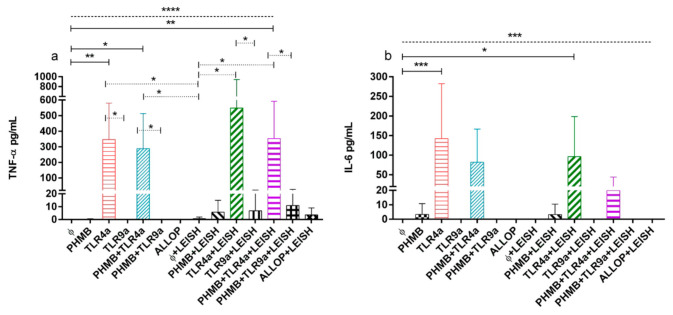
(**a**) TNF-α and (**b**) IL-6 concentrations from uninfected and infected macrophages treated with the following conditions. Medium alone (Ø), Polyhexamethylene biguanide alone (PHMB), MPLA Toll-like receptor agonist 4 (TLR4a), PHMB *plus* TLR4a (PHMB + TLR4a), CpG ODN Toll-like receptor agonist 9 (TLR9a), PHMB *plus* TLR9a (PHMB + TLR9a), and allopurinol (ALLOP). All the conditions indicated with LEISH are *L. infantum* infected replicates. Significant results are described as * 0.05 > *p* > 0.01, ** 0.01 > *p* > 0.001, *** 0.001 > *p* > 0.0001, **** 0.0001 > *p.* Lines in graphic represent ANOVA (dashed lines), Dunnett´s test (solid lines), and Wilcoxon signed-rank test (dotted lines).

Production of proinflammatory TNF-α and IL-6 cytokines of infected and uninfected cells treated with the different conditions is plotted in Figure 4a,b. TNF-α production in uninfected macrophages without treatment was significantly lower than the rest of the treatments with the exception of PHMB alone, TLR9a, PHMB + TLR9a, and allopurinol (ANOVA: *p <* 0.0001). Uninfected macrophages controls secreted lower TNF-α than uninfected cells treated with TLR4a alone (Dunnett’s test: *p =* 0.0017) and with PHMB in combination with TLR4a (Dunnett’s test: *p =* 0.0134); and when compared to infected cells treated with PHMB complex with TLR4a (Dunnett’s test: *p =* 0.0014) (Figure 4a). 

In addition, TNF-α concentrations of non-treated and infected macrophages were significantly lower than uninfected cells treated with TLR4a alone or in combination with PHMB (Wilcoxon signed-rank test: *p =* 0.043). Moreover, infected macrophages alone secreted lower TNF-α concentrations when compared to infected cells treated with TLR4a alone or in combination with PHMB (Wilcoxon signed-rank test: *p =* 0.043). Furthermore, infected macrophages without treatment produced lower TNF-α concentrations than infected cells treated with TLR4a alone or associated to PHMB (Wilcoxon signed-rank test: *p =* 0.043). In addition, infected and uninfected macrophages treated with TLR4a were significantly more potent on production of TNF-α than TLR9a, either associated or not with PHMB (Wilcoxon signed-rank test: *p =* 0.043) (Figure 4a).

Uninfected macrophages without treatment produced lower significant levels of IL-6 when compared to the rest of the conditions (ANOVA: *p <* 0.0001), particularly when compared to uninfected macrophages treated with TLR4a alone (Dunnett’s test: *p =* 0.0002) and infected macrophages treated with TLR4a alone (Dunnett’s test: *p =* 0.0228) (Figure 4b).

At 24 h, a trend of higher IL-6 concentration was detected after TLR4a treatment in complex or not with PHMB, on either infected or uninfected cells. In addition, only a trend of higher concentration of IL-6 was observed on infected and uninfected cells treated with TLR4a when compared with TLR9a, in complex or not with PHMB.

## 4. Discussion

To the best of our knowledge, this study is the first to demonstrate the susceptibility of the *L. infantum* promastigote and amastigote forms to PHMB. In addition, the susceptibility of the *L. infantum* strain to conventional drugs, such as miltefosine and allopurinol was corroborated [36]. Furthermore, the study evaluated the use of TLR ligands as polyplex nanoparticles with PHMB, observing a potent antiparasitic and proinflammatory effect in combination with TLR4a. 

The susceptibility of promastigotes to conventional antileishmanial drugs was determined. As expected, the IC_50_ value obtained for miltefosine (9.455 µM) was in the range determined by others [36]. Allopurinol IC_50_ value (1.495 µM) was also in the range registered by one group [36], but also lower than other studies [37,38,39]. Several studies recommended to avoid the use of miltefosine as monotherapy, and have been encouraged to advocate for its use in combined therapies in order to evade drug resistance [6,36,40]. Additionally, a previous *in vitro* study involving different strains of *Leishmania* parasite remarked how easily *Leishmania* parasite adapts to allopurinol [36]. The genetic characteristics of miltefosine resistance in human patients with leishmaniosis have been described [41,42]. Unfortunately, less conclusive studies are available related to miltefosine resistance in CanL [36,43]. Regarding allopurinol, resistance has been largely investigated in CanL [37,38,39].

Here, we studied the antileishmanial effect of PHMB, a compound not investigated as a treatment for *L. infantum*. However, a previous study demonstrated its properties against *L. major* [34]. In addition, the same study determined an IC_50_ value PHMB concentration of 0.41 µM for *L. major* promastigotes [34], which was lower but similar to that observed in our study for *L. infantum* promastigotes (1.495 µM). In this case, diverse drug sensitivities encountered with each *Leishmania* species might be the cause of such minor difference observed when IC_50_ values were compared. 

The mechanism(s) of action of PHMB remain unclear. However, a previous study demonstrated that this polymer preferentially disrupts microbial membranes, specifically interacting with phospholipid acids [44]. Recently, it was indicated that *Acanthamoeba castellanii* trophozoites and cysts exposed to PHMB presented cell shrinkage and membrane blebbing, which is a hallmark for apoptosis [45]. Firdessa et al. [34] observed that *L. major* incubated with PHMB displayed chromosome disruption, consistent with chromosome effects observed in bacteria [32], indicating that multiple mechanisms are likely involved. It is likely that similar mechanisms of action of PHMB are valid for *L. infantum* investigated in our study.

PHMB displayed low cell toxicity in DH-82 canine macrophages (IC_50_ = 6.13 µM) which was at a similar IC_50_ range when compared to other cell lines studied before, such as bone marrow-derived macrophage (BMDM) from BALBc mice (IC_50_ = 4 µM) and 293T kidney epithelial cells(IC_50_ = 26 µM) [34]. In accordance with a previous study, the conventional drugs miltefosine and allopurinol were also minimally cytotoxic [46] with higher IC_50_ concentrations than the limit of detection of the experiments here performed (Table 2). However, a recent study concluded that cytotoxicity of antileishmanial drugs depends on the type of macrophage source under investigation [47].

Our principal goal was to probe the *in vitro* antileishmanial properties of PHMB alone and in polyplex nanoparticles with TLR4a or TLR9a. As discussed in the introduction, TLRs have key roles in the induction of immunological responses to infection, so combinations of antileishmanial drugs and TLR agonists as adjuvant might have a synergic effect. For this purpose, we measured the infection rate and proinflammatory cytokines production after different combination treatments using an amastigote-infected macrophage system. The amastigote-infected macrophage system has been widely demonstrated to be correlated with the clinical response to *Leishmania* infection when testing drug performance [46,47,48,49,50,51]. The leishmanicidal effect of PHMB on *L. infantum* was demonstrated in our study by evaluating the response to several treatments. When compared to untreated cells, we detected a lower percentage of infection in cells exposed to PHMB and less, but not statistically significant intensity of infection. In agreement with results from *L. major in vitro* study, we described an intracellular action of PHMB against the *L. infantum* amastigote forms [34]. As expected, percentage, intensity, and index of infection were significantly higher in the cells not receiving any treatment when compared with cells stimulated with TLR4a or TLR9a alone or in complexes with PHMB. 

The contribution of TLR4 during *Leishmania* infection and treatment has been extensively studied, since it is considered to be important for efficient parasite control during innate immune responses [29,52,53,54,55,56]. A previous study performed on whole blood of dogs stimulated with *L. infantum* antigen and different TLRa, demonstrated that TLR4a induced a potent pro-inflammatory cytokine stimulation [53]. Additionally, secretion of proinflammatory cytokines such as TNF-α [54] as well as its genetic expression [57] due to TLR4 activation, has been demonstrated using peripheral blood mononuclear cells (PBMC) from patients with cutaneous leishmaniosis due to *L. braziliensis* infection. In accordance with those results, we show that stimulation with TLR4a alone (or associated with PHMB) induced a higher production of TNF-α when compared to the rest of the treatments including TLR9a alone or associated to PHMB. However, a trend of higher production of IL-6 was observed after stimulation with PHMB alone or associated with TLR4a. 

The critical role of TLR9 during activation of the protective innate immune response has been demonstrated using an *in vitro* and *in vivo* mouse model of *L. infantum* infection, in which TLR9 was associated with neutrophil recruitment together with DC activation to combat parasite invasion [30]. We showed that PHMB association with TLR9a managed to reduce the percentage of infection, the index of infection and intensity of infection, when compared to un-treated infected macrophages. In accordance, the potential immunostimulatory activity of TLR9a such as CpG ODN to control intracellular parasites as *Leishmania* has been already demonstrated [34,58]. Moreover, PHMB can strongly interact with CpG ODN (TLR9a) as previously described [34], and with MPLA-SM (TLR4a) as demonstrated in our work. The immunomodulatory effect of TLRa for treatment and/or prevention of leishmaniosis is a current topic of investigation. As observed in our study, Firdessa et al. [34] concluded that the antileishmanial effect of PHMB might be facilitated due to its ability to deliver immunomodulators as TLRs agonist. Further studies are needed to assess the possible clinical use of PHMB in polyplex nanoparticles with TLR4a or TLR9a or both in human and animal leishmaniosis.

## 5. Conclusions

We demonstrated, for the first time, the antileishmanial properties of PHMB against *L. infantum* parasites. In addition, our results support a crucial role for TLR4a in providing protection against *L. infantum* by enhancing parasite elimination and pro-inflammatory cytokine production. Moreover, PHMB combined with TLR4a shows promise as a potential anti-*L. infantum* drug combination, as well as inducer of proinflammatory response, as demonstrated by decreased infection and increased TNF-α production. Further investigations regarding the use of immunomodulators such as TLRa associated with new antileishmanial compounds such as PHMB, introduces interesting alternatives for the treatment of a wide-spread neglected zoonotic disease using combined antimicrobial and immunomodulatory strategies.

## Figures and Tables

**Table 1:** Nanoparticle formulations and particle population profiles.

TLR	Ligand	Ligand mg/mL	Polymer mg/mL	DLS Values ± Standard Deviation		
				Size [d.nm]	PDI	Count Rate [kcps]
**TLR4**	MPLA-SM	0.092	0.69	166.7 ± 2.49	0.157 ± 0.01	190.1 ± 1.90
**TLR9**	5′tccatgacgttcctgatgct	0.092	1	75.9 ± 0.83	0.101 ± 0.01	216.8 ± 1.00

Abbreviations: DLS: dynamic light scattering; MPLA-SM: monophosphoryl lipid A from *Salmonella minnesota*; PDI: polydispersity index; TLR: toll-like receptor.

**Table 2:** IC_50_ values for all drugs studied for *L. infantum* promastigotes and cytotoxicity for DH-82 macrophages.

Parasites and Cells	Allopurinol µM	Miltefosine µM	PHMB µM
*L. infantum* promastigotes	0.124	9.455	1.495
DH-82 macrophages	>18.3	>122.678	6.133

Abbreviations: PHMB: polyhexamethylene biguanide.
